# Sinonasal Malignancies in Taiwan (2009–2019): Histologic Distribution, Overall Survival, and Temporal Trends in Hematological Versus Nonhematological Tumors

**DOI:** 10.1002/lio2.70613

**Published:** 2026-10-21

**Authors:** Chi‐Hsien Wu, Ching‐Heng Lin, Kai‐Li Liang

**Affiliations:** ^1^ Department of Medical Education Taichung Veterans General Hospital Taichung Taiwan; ^2^ Department of Public Health College of Medicine, Fu Jen Catholic University New Taipei City Taiwan; ^3^ Department of Industrial Engineering and Enterprise Information Tunghai University Taichung Taiwan; ^4^ Institute of Public Health and Community Medicine Research Center National Yang Ming Chiao Tung University Taipei Taiwan; ^5^ Department of Otolaryngology Taichung Veterans General Hospital Taichung Taiwan; ^6^ School of Medicine, National Yang Ming Chiao Tung University Taipei Taiwan; ^7^ School of Medicine, Chung Shan Medical University Taichung Taiwan; ^8^ Department of Post‐Baccalaureate Medicine College of Medicine, National Chung Hsing University Taichung Taiwan

**Keywords:** Epstein–Barr virus (EBV), extranodal NK/T‐cell lymphoma (ENKTL), overall survival, prognostic factors, sinonasal malignancies, squamous cell carcinoma, Taiwan

## Abstract

**Background:**

Sinonasal malignancies are rare and histologically diverse, with limited population‐based data from Asia. This study investigated the histologic distribution, factors associated with overall survival (OS), and temporal survival patterns in Taiwan.

**Methods:**

This retrospective population‐based study used linked data from the Taiwan National Health Insurance Research Database and Taiwan Cancer Registry. Adults with invasive nasal cavity or paranasal sinus malignancies diagnosed between 2009 and 2019 were included. Factors associated with OS were evaluated using multivariable Cox regression analysis separately for hematological and nonhematological malignancies.

**Results:**

Among 1494 patients, 718 (48.1%) had hematological malignancies, and 776 (51.9%) had nonhematological malignancies. Squamous cell carcinoma predominated among nonhematological malignancies, whereas T‐cell and NK/T‐cell lymphomas were more common than mature B‐cell lymphomas among hematological malignancies. Increasing age was associated with a higher hazard of death in both groups. In nonhematological malignancies, recorded surgical procedures were associated with a lower hazard of death (adjusted hazard ratio [aHR], 0.52; 95% CI, 0.42–0.66), whereas chemotherapy was associated with a higher hazard (aHR, 1.81; 95% CI, 1.36–2.39). In hematological malignancies, diagnosis during 2015–2019 was associated with a lower hazard of death than during 2009–2014 (aHR, 0.76; 95% CI, 0.60–0.96).

**Conclusion:**

Sinonasal malignancies in Taiwan showed distinct histologic and survival patterns. The predominance of T‐cell and NK/T‐cell neoplasms and improved survival in the more recent period highlight important epidemiological characteristics of hematological sinonasal malignancies in Taiwan.

**Level of Evidence:**

3.

## 1 Introduction

Sinonasal malignancies are rare tumors, accounting for approximately 5% of all head and neck cancers, with an incidence of approximately 0.5–1.0 per 100,000 individuals [1, 2]. These neoplasms arise within the nasal cavity and paranasal sinuses and encompass a broad spectrum of histologic entities, including epithelial malignancies, sarcomas, mucosal melanomas, olfactory neuroblastomas, and sinonasal lymphomas [3]. Common presenting symptoms include nasal obstruction, facial pressure, and epistaxis. These symptoms frequently overlap with those of benign inflammatory sinonasal disorders, potentially contributing to a delayed diagnosis [1, 4].

The epidemiology of sinonasal malignancies varies geographically, and these differences are particularly pronounced in sinonasal lymphomas. In Asian populations, extranodal natural killer/T‐cell lymphoma (ENKTL), nasal type, represents a major subtype of sinonasal lymphoma and predominantly involves the nasal cavity. In contrast, B‐cell lymphomas, particularly diffuse large B‐cell lymphoma (DLBCL), are more frequently encountered in Western populations and more commonly involve the paranasal sinuses [4, 5, 6]. These geographic differences are clinically relevant because ENKTL and DLBCL differ substantially in their pathogenesis, anatomic distribution, treatment, and prognosis. ENKTL is strongly associated with Epstein–Barr virus (EBV) and is characterized by an aggressive clinical course, whereas sinonasal DLBCL generally demonstrates greater responsiveness to rituximab‐based systemic therapy [7, 8, 9]. Variations in EBV epidemiology, host genetic susceptibility, and environmental exposures may contribute to geographic heterogeneity in lymphoma subtype distribution [5, 7, 9].

Consequently, epidemiological findings derived predominantly from Western populations may not fully represent the disease spectrum and outcomes of sinonasal malignancies in Asian populations. This issue is particularly relevant to hematological malignancies, as geographic differences in the distribution of lymphoma subtypes may influence population‐level treatment patterns and survival outcomes. Despite these regional differences, large‐scale population‐based studies describing the epidemiology, long‐term outcomes, and temporal trends of sinonasal malignancies in Asian populations are limited.

Accurate diagnosis and treatment of sinonasal malignancies require an integrated approach incorporating histopathology, immunophenotyping, serological testing, such as EBV assessment, and advanced imaging [4, 7]. Advances in surgical techniques, diagnostic strategies, radiotherapy, and systemic therapies have contributed to evolving treatment paradigms and improved outcomes [1, 10, 11]. However, because treatment strategies and prognosis vary considerably across histologic categories, region‐specific real‐world data are needed to better characterize these heterogeneous malignancies.

Therefore, this nationwide population‐based study aimed to investigate the epidemiology, survival outcomes, and prognostic factors of sinonasal malignancies in Taiwan. Particular attention was given to the differences between hematological and nonhematological malignancies, given the marked geographic variation in the distribution of sinonasal lymphoma in Asian populations [12, 13, 14].

## 2 Materials and Methods

### 2.1 Study Design and Data Sources

This retrospective population‐based study used linked data from Taiwan's National Health Insurance Research Database (NHIRD) and the Taiwan Cancer Registry (TCR). Adult patients with invasive sinonasal malignancies diagnosed between 2009 and 2019 were identified using International Classification of Diseases for Oncology, Third Edition (ICD‐O‐3) topography codes for the nasal cavity (C30.0) and paranasal sinuses (C31.x) [12, 13, 14]. Patients aged < 18 years were excluded. Demographic characteristics, comorbidities, treatment‐related variables, and survival outcomes were obtained from the linked databases.

### 2.2 Grouped Analysis of Sinonasal Malignancies

Sinonasal malignancies were classified as hematological or nonhematological neoplasms according to the histology/behavior (HISTBEH) variable derived from ICD‐O‐3 morphology coding in the TCR. Because several individual histologic subtypes were uncommon, tumors were aggregated for analysis according to the first two digits of the recorded morphology code. This grouping also facilitated compliance with data privacy and disclosure‐control requirements restricting the reporting of categories with small case numbers. Therefore, these two‐digit categories represent analytical groupings of related morphology codes rather than individual histologic diagnoses. For example, Group 80 comprised epithelial malignancies with morphology codes beginning with “80,” predominantly squamous cell carcinoma, but also including sinonasal undifferentiated carcinoma and NUT carcinoma. Group 95 was heterogeneous and included olfactory neuroblastoma, as well as a small number of nonspecific lymphoma diagnoses. The detailed histologic composition of each group is presented in Table 1.

**TABLE 1:** Histological characteristics of sinonasal malignancies stratified with hematological or nonhematological malignancies based on the first two digits of the histology behavior grouping classification.

Characteristic	Sinonasal malignancies		Total, *N*
	Nonhematological (*n* = 776)	Hematological (*n* = 718)	
	*N*	*N*	
Histology behavior grouping			
80: Epithelial neoplasms: squamous cell carcinoma; sinonasal undifferentiated carcinoma; NUT carcinoma	459	0	459
81: Adenocarcinoma, intestinal type; basaloid carcinoma	37	0	37
82: Adenoid cystic carcinoma; non‐intestinal type adenocarcinoma	83	0	83
83: Clear cell carcinoma; adnexal carcinoma	3	0	3
84: Sebaceous adenocarcinoma; mucinous adenocarcinoma	6	0	6
85: Adenosquamous carcinoma; epithelial–myoepithelial carcinoma; acinic cell carcinoma	11	0	11
87: Sinonasal mucosal melanoma	51	0	51
88: Fibrosarcoma, NOS; hemangiopericytoma; malignant fibrous histiocytoma; leiomyosarcoma, NOS malignant fibrous histiocytoma	15	0	15
89: Rhabdomyosarcoma	21	0	21
90: Biphenotypic sinonasal sarcoma; teratoma, malignant, NOS	8	0	8
91: Angiosarcoma; Kaposi sarcoma; osteosarcoma, NOS	7	0	7
92 + 93: Chondrosarcoma; Ewing sarcoma; odontogenic malignancies; peripheral neuroectodermal tumor	5	0	5
95 Olfactory neuroblastoma; malignant lymphoma, NOS; B‐cell lymphoma, NOS	70	8	78
96 Mature B‐cell lymphomas including diffuse large B‐cell lymphoma (DLBCL), NOS	0	270	270
97: Extranodal NK/T‐cell lymphoma, nasal type; peripheral T‐cell lymphoma; precursor T‐cell lymphoblastic lymphoma	0	433	433
98 + 99: Myeloid sarcoma; extramedullary involvement of leukemias	0	7	7

Abbreviation: NOS, not otherwise specified.

### 2.3 Demographic Characteristics, Comorbidities and Treatment‐Related Variables

Baseline variables included age at diagnosis (< 50, 50–59, 60–69, 70–79, and ≥ 80 years), sex, and comorbidities, including diabetes mellitus, hypertension, and hyperlipidemia, which were identified using ICD‐9‐CM and ICD‐10‐CM codes. Treatment‐related procedures, including surgical procedures, chemotherapy, and radiotherapy, were identified using procedure codes and reimbursement records from the NHIRD. Because claims‐based data did not permit reliable differentiation between diagnostic tissue‐acquisition procedures and definitive oncologic resection, the surgical procedure variable was interpreted as the presence of a recorded surgical procedure rather than specifically as therapeutic tumor resection.

### 2.4 Outcome Measures

Survival analyses were conducted based on the calculation of overall survival (OS), defined as the time from the initial diagnosis of sinonasal malignancy to death from any cause. Patients were censored at the end of the follow‐up period or when they withdrew from the insurance program.

### 2.5 Statistical Analysis

Multivariable Cox proportional hazards regression models were applied separately for nonhematological and hematological sinonasal malignancies to identify independent prognostic factors for OS. Adjusted hazard ratios (aHRs) with 95% confidence intervals (CIs) were reported. A two‐sided *p*‐value < 0.05 was considered statistically significant. Statistical analyses were performed using SAS software.

## 3 Results

### 3.1 Histological Distribution of Sinonasal Malignancies

The histological distribution of sinonasal malignancies according to the predefined ICD‐O‐3 morphology groupings is shown in Table 1. Among the 776 nonhematological malignancies, Group 80 epithelial malignancies were the most common (*n* = 459, 59.1%), predominantly comprising squamous cell carcinoma and related epithelial tumor. Groups 81–85, comprising glandular and other epithelial malignancies, accounted for 140 cases, of which Group 82 (including adenoid cystic carcinoma and nonintestinal‐type adenocarcinoma) was the largest subgroup (*n* = 83). Sinonasal mucosal melanoma (Group 87) accounted for 51 cases, whereas sarcoma‐related groups accounted for 56 cases. Group 95 included 70 patients with olfactory neuroblastomas in the nonhematological cohort and eight patients with nonspecific lymphoma diagnoses in the hematological cohort. Among hematological malignancies, mature B‐cell lymphomas (Group 96) accounted for 270 cases, whereas Group 97, comprising extranodal NK/T‐cell lymphoma, peripheral T‐cell lymphoma, and precursor T‐cell lymphoblastic lymphoma, accounted for 433 cases.

### 3.2 Patient Characteristics

A total of 1508 patients with sinonasal malignancies were initially identified. After excluding 14 patients aged < 18 years, 1494 patients were included in the final cohort. Of these, 718 (48.1%) had hematological malignancies and 776 (51.9%) had nonhematological malignancies. Baseline characteristics are summarized in Table 2. Overall, 24.2% of patients were aged < 50 years, whereas 53.1% were aged ≥ 60 years, and 63.3% were male. Diabetes mellitus, hypertension, and hyperlipidemia were present in 27.0%, 47.9%, and 35.7% of patients, respectively.

**TABLE 2:** Characteristics of patients with sinonasal malignancy retrieved from the Taiwan Cancer Registry.

Characteristics	Sinonasal malignancy (*n* = 1494)	
	*n*	%
Age		
< 50	361	24.2
50–59	340	22.8
60–69	336	22.5
70–79	247	16.5
≥ 80	210	14.1
Gender		
Female	548	36.7
Male	946	63.3
Hematological neoplasms		
No	776	51.9
Yes	718	48.1
Diabetes mellitus		
No	1091	73.0
Yes	403	27.0
Hypertension		
No	779	52.1
Yes	715	47.9
Hyperlipidemia		
No	960	64.3
Yes	534	35.7
Operation		
No	764	51.1
Yes	730	48.9
Radiation therapy		
No	970	64.9
Yes	524	35.1
Chemotherapy		
No	644	43.1
Yes	850	56.9
Death		
No	813	54.4
Yes	681	45.6

### 3.3 Multivariable Analysis of Factors Associated With OS

Table 3 presents the multivariable Cox proportional hazards regression analyses stratified by hematological versus nonhematological malignancies. Among patients with nonhematological malignancies (*n* = 776), increasing age was associated with a progressively higher hazard of death. Compared with patients aged < 50 years, the aHRs were 1.87 (95% CI, 1.30–2.70; *p* = 0.001) for those aged 60–69 years, 2.82 (95% CI, 1.88–4.23; *p* < 0.001) for those aged 70–79 years, and 4.47 (95% CI, 2.86–6.98; *p* < 0.001) for those aged ≥ 80 years. Male sex was also associated with a higher hazard of death (aHR, 1.52; 95% CI, 1.18–1.95; *p* = 0.001), whereas diabetes mellitus, hypertension, and hyperlipidemia were not significantly associated with OS. Recorded surgical procedures were associated with a lower hazard of death (aHR, 0.52; 95% CI, 0.42–0.66; *p* < 0.001). Radiotherapy was not significantly associated with OS (aHR, 1.22; 95% CI, 0.94–1.59; *p* = 0.139), whereas chemotherapy was associated with a higher hazard of death (aHR, 1.81; 95% CI, 1.36–2.39; *p* < 0.001).

**TABLE 3:** Multivariable analysis of factors associated with overall survival of sinonasal malignancies.

Variables	Sinonasal malignancy							
	Non‐hematological (*n* = 776)				Hematological (*n* = 718)			
	aHR	95% CI		*p*	aHR	95% CI		*p*
Age								
< 50	1.00				1.00			
50–59	1.17	0.82	1.68	0.386	1.24	0.83	1.85	0.290
60–69	1.87	1.30	2.70	0.001	1.72	1.15	2.58	0.009
70–79	2.82	1.88	4.23	< 0.001*	2.31	1.50	3.57	< 0.001*
≥ 80	4.47	2.86	6.98	< 0.001*	4.94	3.18	7.68	< 0.001*
Gender								
Female	1.00				1.00			
Male	1.52	1.18	1.95	0.001*	0.97	0.78	1.22	0.813
Diabetes mellitus								
No	1.00				1.00			
Yes	1.09	0.84	1.41	0.519	1.24	0.96	1.60	0.106
Hypertension								
No	1.00				1.00			
Yes	0.96	0.74	1.25	0.756	0.89	0.68	1.17	0.394
Hyperlipidemia								
No	1.00				1.00			
Yes	0.79	0.61	1.02	0.071	0.85	0.66	1.09	0.204
Operation								
No	1.00				1.00			
Yes	0.52	0.42	0.66	< 0.001*	0.79	0.61	1.02	0.067
Chemotherapy								
No	1.00				1.00			
Yes	1.81	1.36	2.39	< 0.001*	1.11	0.85	1.45	0.459
Radiation therapy								
No	1.00				1.00			
Yes	1.22	0.94	1.59	0.139	1.18	0.92	1.53	0.194

* *p* < 0.05.

Among patients with hematological malignancies (*n* = 718), increasing age was similarly associated with a higher hazard of death. Compared with patients aged < 50 years, the aHRs were 1.72 (95% CI, 1.15–2.58; *p* = 0.009), 2.31 (95% CI, 1.50–3.57; *p* < 0.001), and 4.94 (95% CI, 3.18–7.68; *p* < 0.001) for patients aged 60–69, 70–79, and ≥ 80 years, respectively. Sex and metabolic comorbidities were not significantly associated with OS. Recorded surgical procedures were not significantly associated with OS (aHR, 0.79; 95% CI, 0.61–1.02; *p* = 0.067), nor were chemotherapy (aHR, 1.11; 95% CI, 0.85–1.45; *p* = 0.459) or radiotherapy (aHR, 1.18; 95% CI, 0.92–1.53; *p* = 0.194).

### 3.4 Prognostic Analysis by Histologic Group and Diagnostic Period

Table 4 presents the histology‐specific analyses, using Group 80 as the reference category for nonhematological malignancies and Group 95 as the reference category for hematological malignancies. Among nonhematological malignancies, Group 88 sarcomas were associated with a higher hazard of death than Group 80 epithelial malignancies (aHR, 2.48; 95% CI, 1.30–4.73; *p* = 0.006), as was sinonasal mucosal melanoma (Group 87; aHR, 1.89; 95% CI, 1.32–2.72; *p* = 0.001). In contrast, Group 82, comprising predominantly adenoid cystic carcinoma and nonintestinal‐type adenocarcinoma, was associated with a lower hazard of death (aHR, 0.62; 95% CI, 0.39–0.97; *p* = 0.037). No statistically significant differences were observed among the hematological histological groups when compared with Group 95.

**TABLE 4:** Prognostic analysis by histology (M code) in overall survival of sinonasal malignancies.

Variables	Sinonasal malignancies							
	Non‐hematological (*n* = 776)				Hematological (*n* = 718)			
	aHR	95% CI		*p*	aHR	95% CI		*p*
M‐code (HISTBEH)								
80: Epithelial neoplasms: squamous cell carcinoma, sinonasal undifferentiated carcinoma, NUT carcinoma	1.00							
81: adenocarcinoma, intestinal type; basaloid carcinoma	0.88	0.51	1.53	0.658				
82: adenoid cystic carcinoma; nonintestinal type adenocarcinoma	0.62	0.39	0.97	0.037*				
83: Clear cell carcinoma, adnexal carcinoma	0.72	0.10	5.27	0.749				
84: Sebaceous adenocarcinoma; mucinous adenocarcinoma	0.60	0.19	1.95	0.398				
85: Adenosquamous carcinoma; epithelial–myoepithelial carcinoma; acinic cell carcinoma	1.42	0.58	3.48	0.439				
87: Sinonasal mucosal melanoma	1.89	1.32	2.72	0.001*				
88: Fibrosarcoma, NOS; hemangiopericytoma; malignant fibrous histiocytoma; leiomyosarcoma, NOS; malignant fibrous histiocytoma	2.48	1.30	4.73	0.006*				
89: Rhabdomyosarcoma	0.78	0.41	1.50	0.455				
90: Biphenotypic sinonasal sarcoma; teratoma, malignant, NOS	2.18	0.79	5.98	0.130				
91: Angiosarcoma; Kaposi sarcoma; osteosarcoma, NOS	1.03	0.32	3.26	0.967				
92 + 93: Chondrosarcoma; Ewing sarcoma; odontogenic malignancies; peripheral neuroectodermal tumor	1.83	0.57	5.89	0.311				
95 Olfactory neuroblastoma; malignant lymphoma, NOS; B‐cell lymphoma, NOS	0.64	0.38	1.09	0.101	1.00			
96 Mature B‐cell lymphomas including diffuse large B‐cell lymphoma (DLBCL), NOS					0.73	0.26	2.05	0.549
97: Extranodal NK/T‐cell lymphoma, nasal type; peripheral T‐cell lymphoma; precursor T‐cell lymphoblastic lymphoma					0.99	0.36	2.72	0.991
98 + 99: Myeloid sarcoma; extramedullary involvement of leukemias					1.94	0.47	8.03	0.362

Abbreviation: aHR, adjusted hazard ratio.

* *p* < 0.05.

The temporal survival analyses are shown in Table 5. Compared with patients diagnosed between 2009 and 2014, those with hematological malignancies diagnosed between 2015 and 2019 had a lower hazard of death (aHR, 0.76; 95% CI, 0.60–0.96; *p* = 0.021). No statistically significant difference was observed between the two diagnostic periods among patients with nonhematological malignancies (aHR, 0.89; 95% CI, 0.69–1.15; *p* = 0.365).

**TABLE 5:** Prognostic analyses by time period.

Time period	Sinonasal malignancies							
	Nonhematological (*n* = 776)				Hematological (*n* = 718)			
	aHR	95% CI		*p*	aHR	95% CI		*p*
Year								
2009–2014	1.00				1.00			
2015–2019	0.89	0.69	1.15	0.365	0.76	0.60	0.96	0.021*

Abbreviation: aHR, adjusted hazard ratio.

* *p* < 0.05.

## 4 Discussion

Sinonasal malignancies are a heterogeneous group of tumors with diverse histological origins and clinical behaviors [15]. Using a nationwide population‐based dataset, this study provides a comprehensive overview of the epidemiology, histological distribution, and prognostic factors of sinonasal malignancies in Taiwan. Several clinically important findings emerged from our analysis. First, the histological distribution of sinonasal malignancies in Taiwan was largely comparable to that in previous international studies: heterogeneous histological subtypes, and T‐cell lymphoma was more prevalent than B‐cell lymphoma, as in other Asian countries [2]. Second, increasing age was consistently associated with poorer survival outcomes in both hematological and nonhematological malignancies. Third, surgical intervention remains an important prognostic factor for nonhematological tumors. Most importantly, patients with hematological sinonasal malignancies diagnosed between 2015 and 2019 had a significantly lower hazard of death than those diagnosed between 2009 and 2014. This temporal difference may partly reflect advances in multidisciplinary management, including contemporary systemic therapy and radiotherapy techniques, although treatment‐related explanations cannot be established from the present observational data [8, 10, 16].

Among patients with nonhematological sinonasal malignancies, advanced age was independently associated with poor OS. Recorded surgical procedures were associated with a lower hazard of death. This finding is broadly consistent with the central role of surgical resection in the management of many resectable nonhematological sinonasal malignancies. However, the observed association should not be interpreted as a causal effect of surgery because information on tumor stage, resectability, surgical intent, and margin status was unavailable [1]. Conversely, chemotherapy was associated with a higher hazard of death. This finding should not be interpreted as a detrimental effect of chemotherapy but more likely reflects confounding by indication, as patients receiving systemic therapy may have had more advanced, unresectable, or biologically aggressive disease.

Histology‐specific analyses also demonstrated substantial heterogeneity among nonhematological tumors. Squamous cell carcinoma is the predominant subtype. Previous studies have reported that adenoid cystic carcinoma generally follows a more indolent clinical course despite its tendency for late recurrence, whereas sinonasal mucosal melanoma is associated with particularly poor outcomes because of its aggressive biological behavior and high metastatic potential [1, 6]. Overall, the histologic distribution and prognostic characteristics of nonhematological sinonasal malignancies in Taiwan were comparable to those reported in Western populations.

In contrast, hematological sinonasal malignancies in Taiwan demonstrate a distinct subtype distribution characterized by a high prevalence of aggressive T‐cell and NK‐cell malignancies, including peripheral T‐cell lymphoma, ENKTL, and precursor T‐cell lymphoma, consistent with the known epidemiology of Epstein–Barr virus‐associated lymphoid neoplasms in East Asia [5, 7, 9, 17]. Among this group of sinonasal malignancies, ENKTL is characterized by angio‐destructive growth, extensive necrosis, and aggressive clinical behavior, which have historically contributed to poorer survival outcomes than those of diffuse large B‐cell lymphoma [7, 8, 9, 16]. Although previous studies have reported poorer outcomes for ENKTL than for DLBCL in some clinical settings [7, 8, 9, 16], our grouped histology analysis did not demonstrate statistically significant survival differences among the hematological morphology groups. This may partly reflect the heterogeneity of the registry‐based groupings and the lack of detailed, disease‐specific clinical information.

One of the notable findings of this study was the lower hazard of death among patients with hematological sinonasal malignancies diagnosed in 2015–2019 than those diagnosed in 2009–2014. During this period, the management of hematological malignancies has evolved substantially, including the increasing use of L‐asparaginase‐based regimens for ENKTL and rituximab‐containing regimens for B‐cell lymphomas [8, 10, 16]. Advances in radiotherapy, including intensity‐modulated radiation therapy, may have also contributed to improved disease control while reducing treatment‐related toxicity. However, because detailed information on systemic regimens, radiotherapy techniques, disease stage, and supportive care was unavailable in the present dataset, the observed temporal survival differences cannot be attributed to any specific therapeutic advancement. Changes in diagnostic practices and multidisciplinary care may also have contributed to this.

Accurate diagnosis is particularly important in sinonasal malignancies because their clinical presentation may mimic chronic rhinosinusitis or other benign inflammatory disorders. This diagnostic challenge is especially relevant to sinonasal lymphoma. In our previous clinical study, adequate tissue sampling frequently required repeated or deep biopsy because superficial specimens may contain predominantly inflammatory or necrotic tissue [18]. Thus, surgical procedures in patients with hematological malignancies may serve a diagnostic rather than therapeutic purpose. Definitive surgical resection is generally not a primary treatment modality for sinonasal hematological malignancies, which are predominantly managed with systemic therapy and/or radiotherapy according to histologic subtype. In the present claims‐based analysis, the surgical procedure variable could not reliably distinguish diagnostic tissue acquisition from definitive oncologic resection. Accordingly, the nonsignificant association between recorded surgical procedures and OS in the hematological cohort (aHR, 0.79; 95% CI, 0.61–1.02) should not be interpreted as evidence of a therapeutic survival benefit from surgery. In contrast, definitive resection remains an important component of treatment for many resectable nonhematological sinonasal malignancies.

The major strength of this study lies in its nationwide population‐based design, which minimizes referral bias and provides real‐world evidence on sinonasal malignancies in Taiwan. By linking national insurance claims with cancer registry data, we were able to examine survival patterns in a relatively rare and histologically heterogeneous group of malignancies that are difficult to study in large single‐center cohorts. Several limitations should nevertheless be acknowledged. First, detailed clinicopathological information, including tumor stage, EBV status, radiotherapy dose and technique, systemic treatment regimens, surgical margin status, and molecular characteristics, was unavailable, limiting adjustment for disease severity and treatment details. Second, treatment selection bias and confounding by indication cannot be excluded because treatment decisions are influenced by tumor characteristics, disease extent, and patient condition. Third, claims‐based procedure coding did not permit reliable distinction between diagnostic tissue‐acquisition procedures and definitive oncologic resection, particularly in patients with hematological malignancies. Fourth, aggregation of rare histologic subtypes according to the first two digits of the morphology code resulted in clinically heterogeneous categories and may have obscured subtype‐specific survival differences. Fifth, the retrospective observational design precludes causal inference regarding associations between treatment, diagnostic period, and survival. Finally, although survival status and censoring information were available from the linked databases, individual follow‐up duration could not be reliably summarized; therefore, the distribution of follow‐up time could not be reported.

## 5 Conclusion

This nationwide population‐based study demonstrated distinct histological distributions and survival patterns between hematological and nonhematological sinonasal malignancies in Taiwan. Nonhematological malignancies showed a heterogeneous distribution dominated by epithelial tumors, whereas hematological malignancies were characterized by a high prevalence of T‐cell and NK/T‐cell neoplasms. Patients with hematological malignancies diagnosed between 2015 and 2019 had a lower hazard of death than those diagnosed between 2009 and 2014, although the factors underlying this temporal difference could not be determined from the available data. These findings provide population‐based evidence of the distinct epidemiological and survival patterns of sinonasal malignancies in Taiwan and highlight the importance of histology‐specific interpretation and management.

## Funding

The authors have nothing to report.

## Conflicts of Interest

The authors declare no conflicts of interest.

## Data Availability

The data that support the findings of this study are available on request from the corresponding author. The data are not publicly available due to privacy or ethical restrictions.
